# Test repositioning for functional assessment of neurological outcome after experimental stroke in mice

**DOI:** 10.1371/journal.pone.0176770

**Published:** 2017-05-04

**Authors:** Macarena Hernández-Jiménez, Carolina Peña-Martínez, María del Carmen Godino, Jaime Díaz-Guzmán, María Ángeles Moro, Ignacio Lizasoain

**Affiliations:** 1 Unidad de Investigación Neurovascular, Departamento de Farmacología, Facultad de Medicina, Universidad Complutense, Madrid, Spain; 2 Instituto de Investigación Hospital 12 de Octubre (i+12) and Instituto Universitario de Investigación en Neuroquímica, Madrid, Spain; 3 Servicio de Neurología, Hospital Universitario 12 de Octubre and Instituto de Investigación Hospital 12 de Octubre (i+12), Madrid, Spain; Indian Institute of Integrative Medicine CSIR, INDIA

## Abstract

Stroke is a cerebrovascular pathology for which the only approved treatment is fibrinolysis. Several studies have focused on the development of new drugs but none has led to effective therapies to date, due, among others, to the difficulty to evaluate clinical deficits in experimental animal models. The present study aims to explore the applicability of known behavioral tests not commonly used in ischemia for the neurological assessment of mice after experimental stroke in different brain areas. A total of 225 CD1 male mice were randomly assigned to permanent middle cerebral artery occlusion by ligature (pMCAO) or permanent anterior cerebral artery occlusion by photothrombosis (pACAO) models. Modified neuroseverity score, footprint test, forced swim test and elevated plus maze were performed. Under these experimental conditions, modified neuroseverity score showed neurological impairment early after experimental stroke in both models. By contrast, the footprint test and the elevated plus maze detected short-term neurological deterioration in the pMCAO model but not in the pACAO model. Furthermore, the forced swim test identified depression-like behavior in mice after ischemia only when the left hemisphere was affected. In conclusion, we propose the repositioning of known neurobehavioral tests, but not commonly used in the stroke field, for the fast detection of neurological impairments early after ischemia, and even specific to discriminate the territory affected by arterial occlusion as well as the hemisphere where brain damage occurs. All these findings may prove useful to improve the experimental design of neuroprotective drugs in order to bridge the gap between experimental studies and clinical trials.

## Introduction

Stroke is one of the main causes of death and disability in developed countries, and it has been classed as a medical emergency. Although numerous studies have attempted the development of new drugs with brain protective effects in experimental models, none has led to effective therapies so far. Therefore, several initiatives have been proposed to overcome the lack of translation of preclinical studies to human clinical trials. Among them, it has been proposed the need to assess the functional response in addition to infarct volume as outcome measures that may help to bridge the gap from animal research to human trials [1].

Indeed, the ultimate goal of stroke treatment is the restoration of behavioral function in patients. Therefore, identifying behavioral deficits in animal stroke models is essential for potential translational applications. In this context, in contrast to the availability of tests for clinical evaluation of patients after stroke, mouse neurobehaviour after cerebral ischemia remains difficult to assess due to the fact that most available tests are adapted from rats [2, 3, 4]. On the other hand, tests commonly used do not reflect the wide variety of human forms of stroke [5]. As a consequence of all these factors, several authors have shown opposite results in the evaluation of deficits after experimental ischemia based on the species, strain, age and experimental model used as well as on the time at which the test is performed [6, 7, 8].

Other aspect to take into account is that brain damage location is crucial to determine both the symptomatology and the prognosis of a stroke. The MCA is the most common site for the occurrence of ischemic stroke. For this reason, the most widely used model of focal ischemia in rodents is the MCAO model that predominantly affects sensory-motor circuits. On the other hand, ACA occlusion results in damage to the frontal lobes producing motor deficits and impairments in learning, memory and executive functions [9]. Although ACA stroke accounts for approximately 2–3% of strokes, the impairments that occur in ACA-brain areas can persist for years and are associated with higher rates of long-term post-stroke disability [10]. Additionally, the effects of a stroke depend on the hemisphere affected. After stroke in the right hemisphere, the left side of the body and functions like vision, memory and anxiety will be affected. Conversely, if the stroke occurs in the left hemisphere, the right side of the body will be affected, producing paralysis on the right side of the body, speech/language problems, and depression. Importantly, assessment of all these important functions is usually overlooked in preclinical stroke research.

All this evidence indicates that the selection of individual tests for each experimental design is crucial for the success of translational stroke research. Since there is a need of novel and sensitive outcome measures with translational applicability, the present study aims to explore the usefulness of known behavioural tests, not commonly used in ischemia, for the neurological assessment of mice after experimental stroke in different brain areas.

## Materials and methods

### Animals

A total of 225 CD1 male mice, 8–10 weeks-old and weighting 25-30g (Harlan, Spain) were used in this study. Animals were housed in ventilated cages at 22°C in a 12h light/dark cycle and 35% humidity with *ad libitum* access to food and water. All testing was performed during the light phase of the cycle.

All procedures were performed in accordance with the European Communities Council Directive (86/609/EEC) and the protocol was approved by the Ethics Committees on Animal Welfare of University Complutense (PROEX 047/16) and were conducted and reported according to ARRIVE guidelines. A special effort was made to reduce the number of animals used in the study and to provide them with the most comfortable conditions possible.

### Surgical procedures

All experiments were performed and quantified in a randomized fashion by investigators blinded to specific conditions for prevention of bias. Two different animal models of ischemic stroke were used to evaluate neurological function in different brain-areas. Permanent middle cerebral artery occlusion by ligature (pMCAO) and permanent anterior cerebral artery occlusion by photothrombosis (pACAO). In both models, animals were anesthetized with isoflurane (IsoVet, Piramal Healthcare, UK), 3% for induction and 1.5% for maintenance, delivered in a 30/70 mixture of O_2_/N_2_O. Body temperature was maintained at 37°C using a homeothermic blanket connected to a rectal probe (HB 101/2, Harvard Apparatus, EE.UU.). The survival rate of the animals until the end of the experiment was 97.5%.

#### Ligature model (pMCAO)

Left common carotid (CCA) and left middle cerebral artery (MCA) were exposed and occluded permanently by ligation as previously described [11]. In another set of experiments, right common carotid and right middle cerebral artery were exposed and ligated. In both, complete interruption of the blood flow was confirmed under an operating microscope. Sham-operated animals were subjected to anesthesia and the surgical procedure but the occlusion of the arteries was omitted. Following surgery, subjects were returned to their cages and allowed free access to water and food.

#### Photothrombotic model (pACAO)

Left or right anterior cerebral artery (ACA) was permanently occluded as described before [12]. After a middle scalp incision, the ACA was identified with a stereomicroscope (PZMIV, World Precision Instruments, EE.UU.). Rose Bengal (Sigma-Aldrich, St. Louis, MO, USA), a photosensitive dye, was dissolved in sterile saline at a concentration of 10 mg/ml, and 10 mg/kg of Rose Bengal was injected intraperitoneally. The brain was illuminated at 549 nm with a cold light lamp (Schot KL1500) for 10 min. Sham-operated animals were subjected to the same surgical procedure except the cold light illumination.

To follow STAIR recommendations [1], before the study we defined some items related to exclusion and endpoint criteria, euthanasia, sample size and the principle of the 3Rs (Replace, Reduction and Refinement). In this way, we used the “Resource Equation Method” [13] to establish sample size resulting in 8–10 animals per experimental group. In order to avoid external influences, surgeries were performed over a year every Tuesday in groups of 8 mice, and 2 animals were allocated to each evaluated behavioral time point. Ischemic animals with infarct volume above 30%IH (n = 7) or under 10%IH (n = 2) were excluded; sham-operated animals with infarct volume > 5%IH (n = 2) were also excluded. We also defined as endpoint criteria those animals who could not move; no animals were included in this category at the end of the study. Finally, animals were sacrificed by cervical dislocation and different organs (brain, blood, liver, muscle, spleen, etc.) were collected and preserved at -80°C for possible future studies.

### Neurobehavioral assessment

For behavioral assessment of mice, different tests were performed at different time points (before surgery and 24h, 48h, 72h or 7 days after stroke). In order to prevent the adaptation to the conditions of the test, each group of mice only performed the test: before surgery (time 0) and another time afterwards. In addition, each group of animals performed a maximum of two different tests in order to avoid possible influences among the various assessments. Mice were randomly distributed in the groups (n = 9–10 per group) and evaluated by two different researches blinded to the surgical procedure.

#### Modified neuroseverity score

Sensory and motor deficits were evaluated through the neuroseverity score adapted for mice. This scale has been used to evaluate neurobehavioral deficits in rats after experimental stroke and in experimental cerebral hemorrhage in mice, and consist of seven different tests that monitor different tasks as described [14]. Here we have added one point more in order to improve the score. Motor score was derived from spontaneous activity, symmetry of limb movements, climbing, balance and coordination. Sensory score was derived from body proprioception, vibrissae, visual and tactile responses.

#### Footprint test

The gait pattern is often evaluated in patients in order to measure chronic hemiparesis due to stroke [15, 16]. In animals, the footprint test has been described to be useful in Parkinson´s and Huntington´s disease but it has rarely been used in experimental stroke. We therefore assessed gait pattern in mice through this test as described [17]. Briefly, the fore and hind feet of the mice were painted with blue and green nontoxic paints, respectively. Animals were then allowed to freely walk along a narrow corridor (50x10x10 cm). Four different gait patterns were analyzed: the stride length, frontbase width, hindbase width and the overlap between front and hind footprints.

#### Forced swim test

To assess depressive-like behavior, mice were forced to swim individually in a glass cylinder filled with water at 25°C. The animals were placed into the cylinder for 6 min and the total duration of their immobility during the last 5 min was recorded [18].

#### Elevated plus maze

Anxiety-like behavior was measured using the elevated plus maze test. The maze consisted in two open and two closed arms which are elevated above the floor 50 cm. Mice were placed individually on the center of the maze and the time spend as well as the number of entries in closed and open arms were recorded during 5 min [19].

### Determination of infarct size

Infarct volume was assessed using magnetic resonance imaging (MRI) [Icon (1T-MRI); Bruker BioSpin GmbH, Ettlingen, Germany] 24 h after surgery. Infarct size was determined as described [20].

### Statistical analysis

Results were expressed as mean±SD for the indicated number of experiments. Prism4 (GraphPad Software, La Jolla, CA) was used for statistical analysis. Unpaired Student t test was used to compare 2 groups. Two-way ANOVA was used to compare >2 groups or parameters with the Bonferroni post hoc test. P<0.05 was considered statistically significant.

## Results

### Exposure to pMCAO or pACAO induce similar infarct volume but in different location

**Fig 1:**
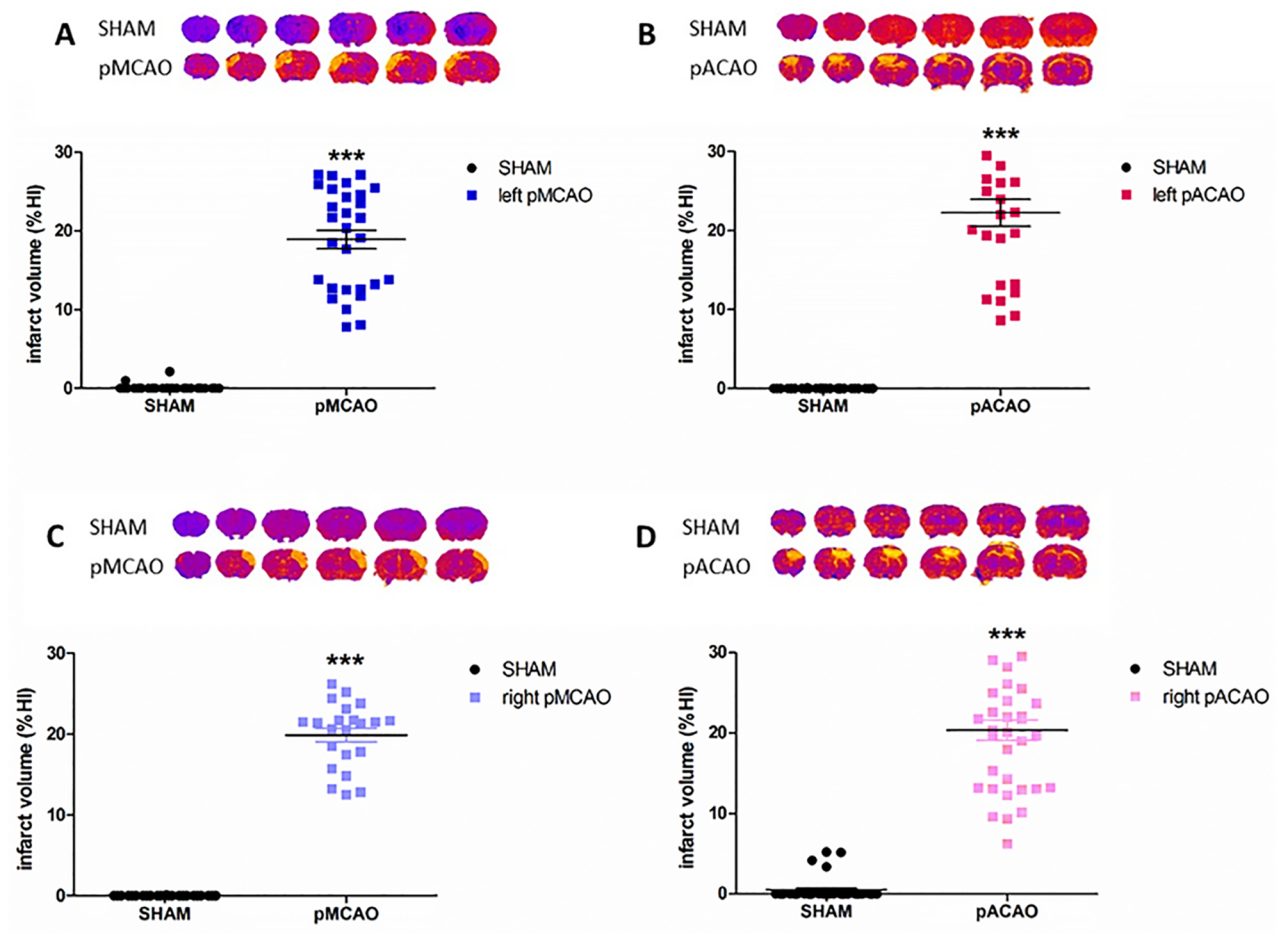
Infarct volume after exposure to pMCAO or pACAO. Quantification of sham and ischemic-mice MRI brain slices 24 h after ischemia. **A**. Left pMCAO (n_SHAM_ = 32, n_pMCAO_ = 29). **B**. Left pACAO (n_SHAM_ = 26, n_pACAO_ = 25). **C**. Right pMCAO (n_SHAM_ = 24, n_pMCAO_ = 22). **D**. right pACAO, (n_SHAM_ = 37, n_pACAO_ = 30). Data are mean±SD (***P<0.001).

Exposure to 8-week-old CD1 mice to permanent MCAO or permanent ACAO induced similar infarct volumes expressed in percentage of infarct hemisphere. After 24 h, left pMCAO resulted in %IH = 18.93 ± 6.40 and left pACAO in %IH = 23.53 ± 8.91 (Fig 1A and 1B). In order to explore the association of several neurological impairments with right or left hemisphere, we performed both models in the contralateral arteries. After occluding right MCA and right ACA, similar infarct volumes were also obtained, as in the opposite hemisphere (pMCAO, %IH = 19.89 ± 4.01; pACAO, %IH = 19.05 ± 6.19) (Fig 1C and 1D). In no case, edema index showed changes between sham-operated and ischemic animals (edema index of left pMCAO = 1,096 ± 0,118; left pACAO = 0,999 ± 0,204; right pMCAO = 1,120 ± 0,191; right pACAO = 1,051 ± 0,085).

As expected [21, 22], the location of the lesioned area was different in the two models. In the pMCAO (right or left) model, lateral surface of frontal lobe and cerebral cortex, capsule, some of the parieto-temporal cortex and cortical layers were affected. While, in the pACAO model, we detected brain damage in medial cerebral cortex, frontal cortex, frontal lobe, olfactory bulb, anterior nucleus accumbens, anterior cingulate, medial septal nuclei and anterior parieto-temporal cortex (Fig 1).

### Modified neuroseverity score is useful to evaluate early neurological impairment after experimental stroke in both models

**Fig 2:**
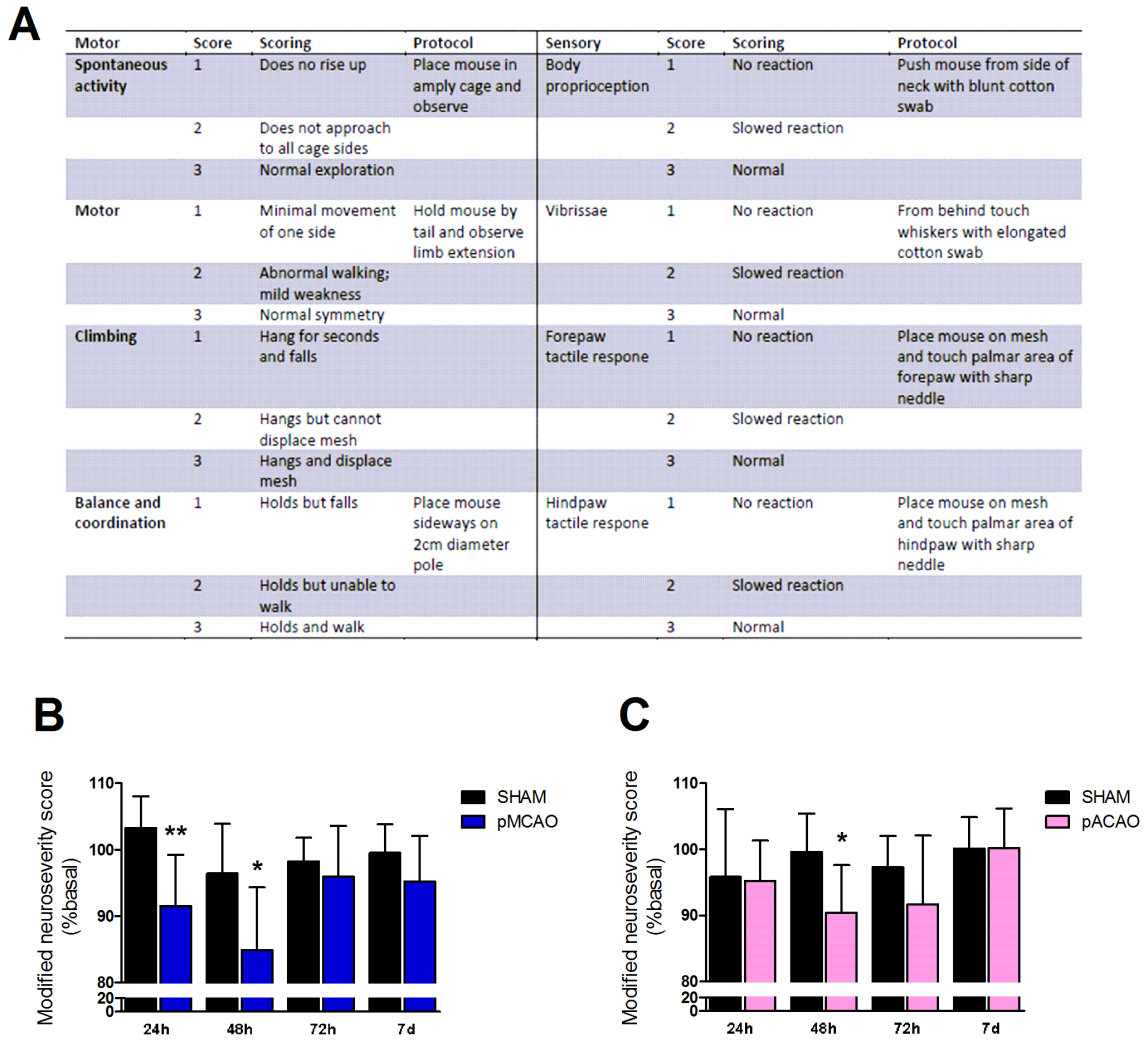
Modified neuroseverity score in pMCAO and pACAO models. Score in modified neuroseverity scale 24 h, 48 h, 72 h and 7 d after surgery in sham-operated animals and mice after pMCAO or pACAO. All data are expressed as percentage of each basal value. **A**. Modified neuroseverity score. B. Score of animals in pMCAO model. **C**. Score after pACAO. Data are mean±SD (n = 9–10; *P<0.05, **P<0.01).

Exposure of mice to pMCAO induced neurological deficits at 24 h and 48 h as determined by the modified neuroseverity score (Fig 2A) when compared with sham-operated animals (n = 9, P<0.05; Fig 2B). Similar results were obtained when ACA was occluded, but only 48 h after ischemia (n = 9, P<0.05; Fig 2C). No differences were shown at other time points studied (72 h and 7 d after ischemia).

### Footprint test is appropriate to measure gait pattern differences in pMCAO model

**Fig 3:**
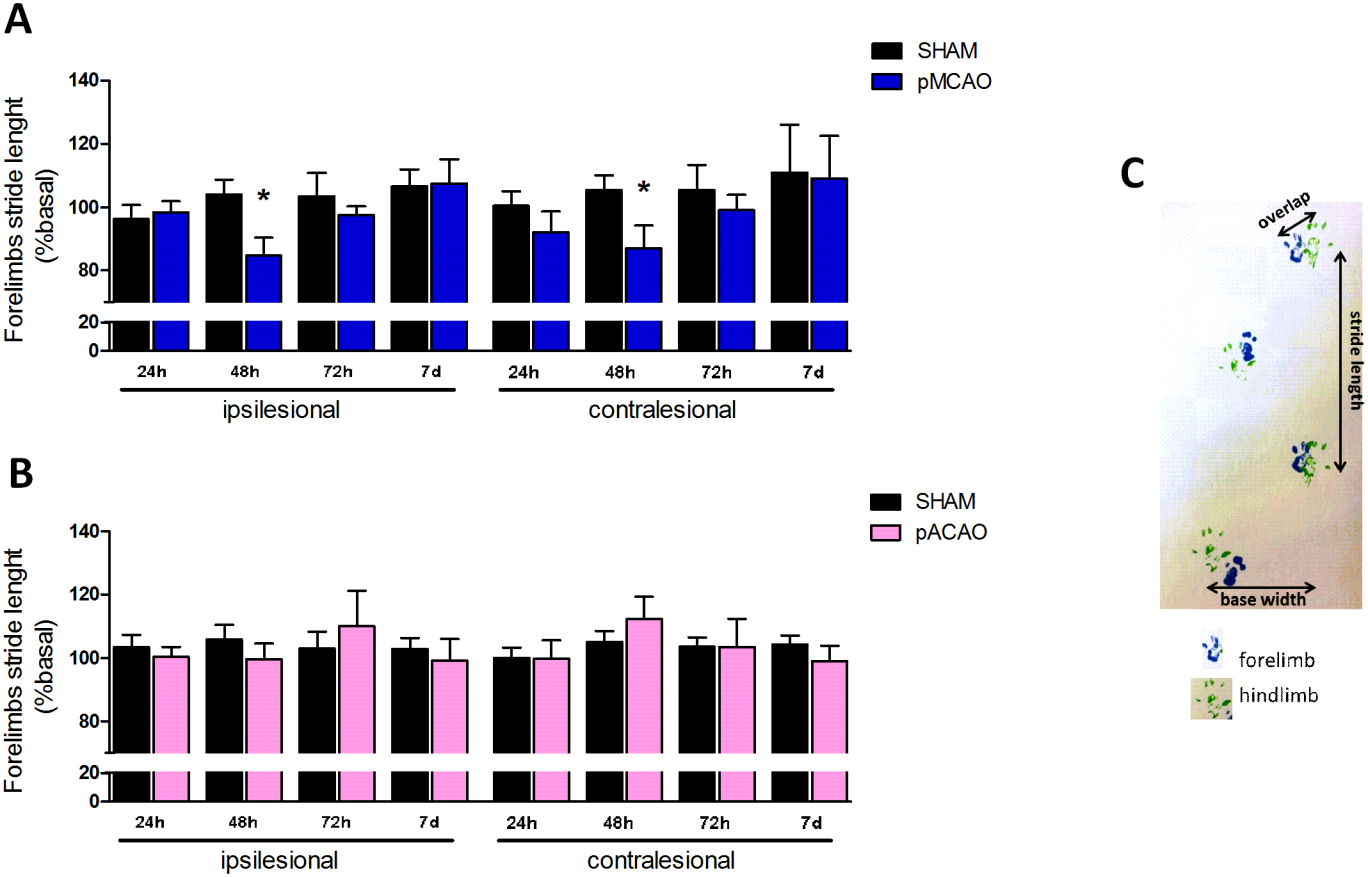
Footprint test after stroke. Quantification of gait pattern 24 h, 48 h, 72 h and 7 d after surgery in sham-operated vs. pMCAO or pACAO mice. All data are expressed as percentage of each basal value. **A**. Distance of ipsilesional and contralesional forelimbs stride length measured in cm after pMCAO. **B.** Distance (cm) of footprint forelimbs stride length after pACAO **C.** Representative walking footprint patterns. Data are mean ± SD (n = 9–10; *P<0.05).

Analysis of the gait pattern in mice after ischemia using the footprint test showed significant deficits in ipsilesional and contralesional forelimb stride length, 48 h after pMCAO vs. the sham group (n = 10, P<0.05; Fig 3A). Other parameters in gait pattern (hindlimb stride length, overlap and front/base base width) did not display any significant differences at any time vs sham-operated animals (n = 10, P<0.05; Table 1).

**Table 1:** Footprint test.

FOOTPRINT TEST			Time after ischemia			
			24h	48h	72h	7d
***pMCAO***	**Hind limb stride length**	**Ipsilesional**	103.0 ± 16.50	103.5 ± 32.0	107.5 ± 13.37	101.2 ± 25.24
		**Contralesional**	97.93 ± 15.49	99.76 ± 22.16	93.13 ± 11.67	110.7 ± 24.13
	**Base width**	**Front**	99.98 ± 18.81	95.14 ± 14.62	97.15 ± 23.31	107.02 ± 28.90
		**Hind**	94.14 ± 19.96	92.79 ± 13.73	91.94 ± 13.41	102.6 ± 22.41
	**Overlap**		0.62 ± 0.28	0.78 ± 0.53	0.69 ± 0.37	0.62 ± 0.43
***pACAO***	**Hind limb stride length**	**Ipsilesional**	95.59 ± 12.36	99.84 ± 17.96	95.10 ± 25.27	94.48 ± 14.52
		**Contralesional**	103.2 ± 14.54	109.1 ± 20.58	108.7 ± 25.75	103.2 ± 10.59
	**Base width**	**Front**	99.60 ± 14.87	102.40 ± 14.62	102.1 ± 15.18	97.88 ± 11.10
		**Hind**	107.2 ± 16.76	106.6 ± 18.96	102.6 ± 20.68	101.3 ± 11.54
	**Overlap**		0.64 ± 0.42	0.61 ± 0.46	0.57 ± 0.42	0.56 ± 0.48

Quantification of ipsi/contralesional hind limb stride length, front/hind base width and overlap, 24h, 48h, 72h and 7 d after pMCAO or pACAO. Data are mean±SD of ischemic mice vs. sham. All data are expressed as the percentage of each respective basal measure except the overlap which is expressed in absolute data.

On the other hand, no differences were detected at any time in the ACAO model (n = 10, P<0.05; Fig 3B and Table 1).

### Elevated plus maze accurately discriminate short-term psychological deficits after experimental cerebral ischemia in the pMCAO model

**Fig 4:**
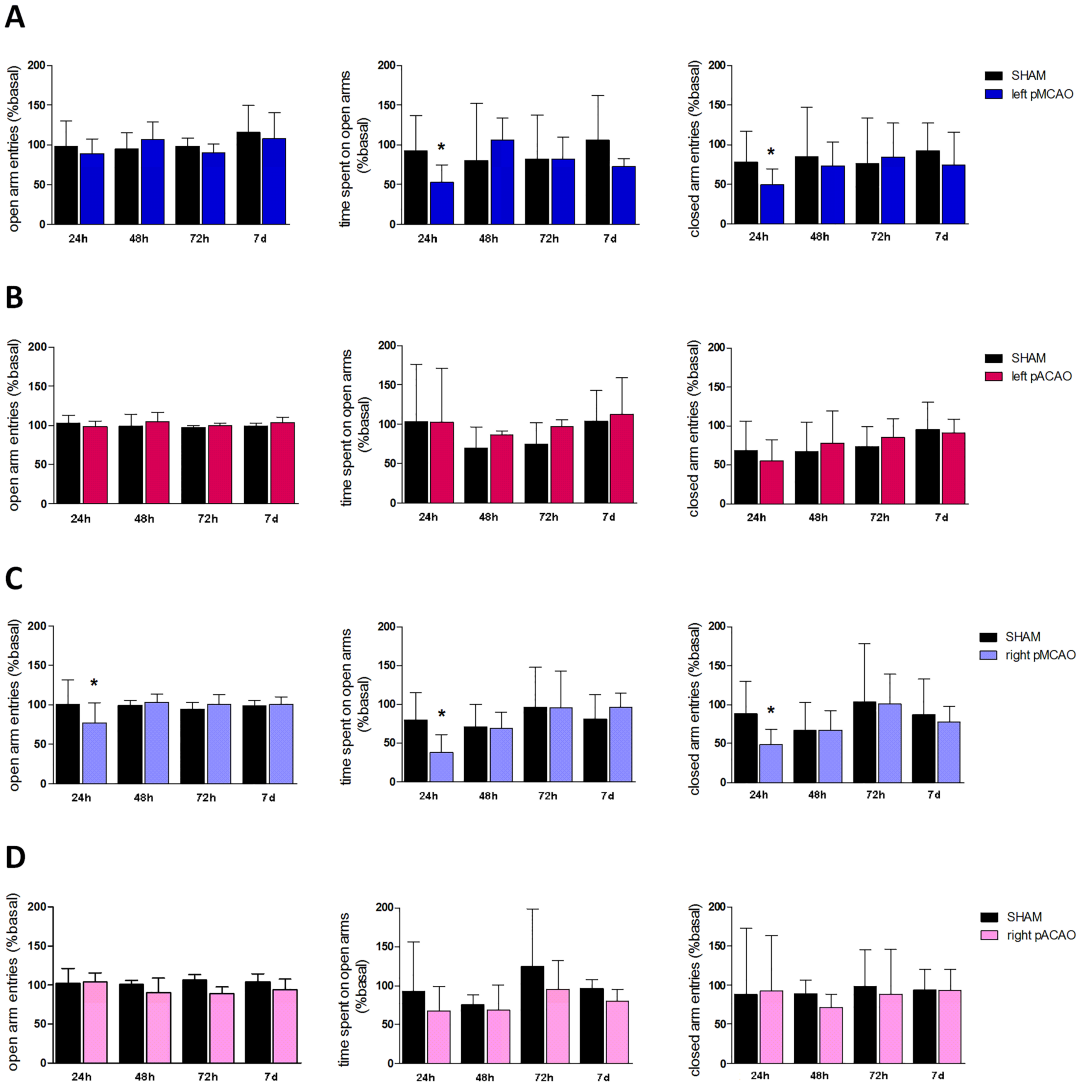
Elevated plus maze after experimental ischemia in mice. Quantification of open arms entries, time spent on open arms and closed arms entries, 24 h, 48 h, 72 h and 7 d after surgery in sham-operated and pMCAO or pACAO animals. All data are expressed as percentage of each basal value. **A.** Left pMCAO model. **B.** Left pACAO model. **C.** Right pMCAO model. **D.** Right pACAO model. Data are mean±SD (n = 9–10; *P<0.05).

In order to detect anxiety-like behavior, we evaluated different parameters in the two models of ischemia using the elevated plus maze test. Specifically, we analyzed time spent on open arms, the number of entries on open arms and number of entries on closed arms. Our results show a decrease in the time spent at the open arms and in the number of entries in the closed arms, 24 h after ischemia, when left MCA was occluded, vs. the sham group (n = 10, P<0.05; Fig 4A). We did not detect any differences after left ACAO in the number of entries either in the open or in the closed arms (n = 10, P<0.05; Fig 4B).

Since some studies have reported an association of anxiety symptoms with right hemispheric lesions in patients [23], we decided to compare the hyperanxious phenotype in mice subjected to ischemia in different hemispheres in both models. In our case, we did not find any significant differences between hemispheres: mice subjected to right MCAO spent less time and reduced the number of entries on open arms, and also showed a decrease in close arms entries 24 h after occlusion (n = 10, P<0.05; Fig 4C); on the other hand, and similarly to left ACAO, right ACA occlusion did not induce any changes when compared with the sham group (n = 10, P<0.05; Fig 4D).

### Forced swim test is a sensitive test to evaluate depressive-like behavior in mice after ischemia only when left hemisphere is affected

**Fig 5:**
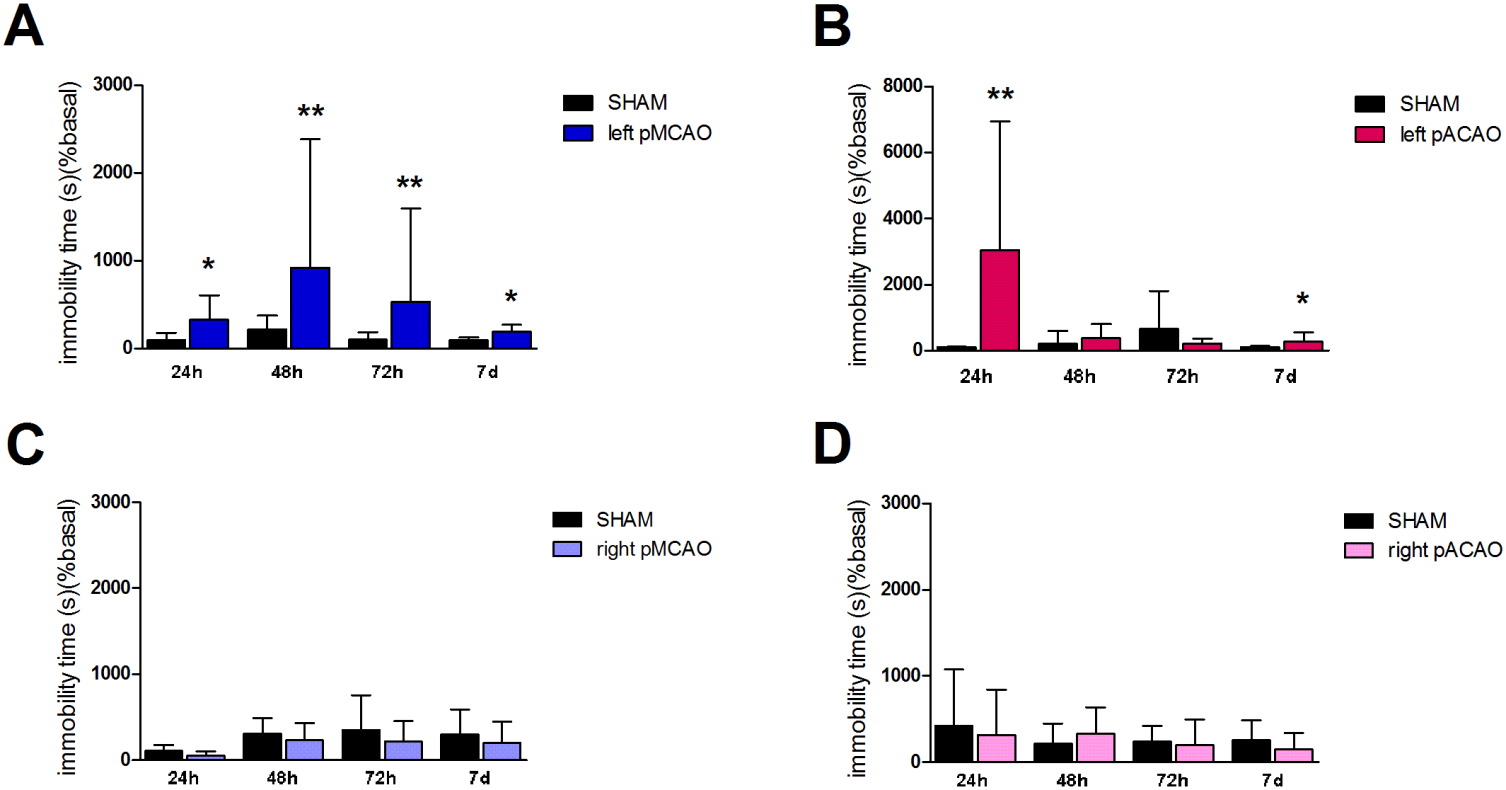
Forced swim test. Quantification of immobility time 24 h, 48 h, 72 h and 7 d after surgery in sham-operated and pMCAO or pACAO animals. All data are expressed as percentage of each basal value. **A.** Left pMCAO. **B.** Left pACAO. **C.** Right pMCAO. **D.** Right pACAO. Data are mean±SD (n = 9–10; *P<0.05).

Depression is a common disorder after stroke. However, depressive symptoms in animal models of stroke are understudied. We therefore explored immobility time in mice after ischemia. Importantly, we detected an increase in immobility time 24 h, 48 h, 72 h and 7 d after left MCA occlusion when compared with sham-operated animals (n = 10, P<0.05; Fig 5A) but not after right MCA occlusion (n = 10, P<0.05; Fig 5C).

Similar results were observed when we occluded left ACA: we found an increase in immobility time at 24 h and 7 d when compared with the corresponding sham group (n = 10, P<0.05; Fig 5B); however, no differences were observed after right ACA occlusion (n = 10, P<0.05; Fig 5D).

## Discussion

Nowadays stroke remains the main cause of disability in developed countries. The lack of translation of the results obtained in experimental pharmacological studies to the clinical practice hinders the development of new neuroprotective drugs. For this reason, assessment of neurological function in rodent models of ischemia has been identified as a major need in the evaluation of drug beneficial-effects in preclinical studies.

We hereby report the applicability of several known neurobehavioral tests, not commonly used in experimental stroke, for a fast assessment of neurobehavioural deficits in the acute phase this disorder. For this purpose, we used CD1 mice in which we evaluated functional deficits early after permanent ischemia in two different brain areas (middle and anterior cerebral arteries regions). We decided to use this mouse strain because it is one of the most commonly used [5] and has the advantage of its low variability in infarct size.

First, we have found that exposure of mice to MCAO by ligature produced somatosensory and motor deficits that were detectable with the modified neuroseverity score at early time points (24 and 48 h after ischemia), whereas in mice exposed to ACAO by photothrombosis, we observed deficits only 48 h after occlusion in this score. Therefore, our results demonstrate that this score is a sensitive test to detect neurobehavioral alterations independent of edema index earlier after ischemia, at least, in CD1 mice and in these two experimental models. This scale -without modification- has been used to evaluate deficits in rats after arterial occlusion with monofilament [14] and after intracerebral hemorrhage in mice [24] but, to our knowledge, its use after permanent cerebral ischemia models in mice has not been reported.

In order to pursue the study of motor deficits, we also employed the footprint test. This test has been typically performed to evaluate gait pattern in experimental mice models of Huntington´s and Parkinson´s diseases [25, 26] and, more recently, motor deficits in rats after experimental spinal cord injury or stroke [27, 28]. Gait is one of the parameters more frequently analyzed in stroke patients [18], so its usefulness would have the additional advantage of its translational potential. Indeed, our results show a significant decrease in both ipsilesional and contralesional forelimb stride length, 48 h after ischemia, in pMCAO model vs. sham-operated animals. This decrease in forelimb stride length in mice has been described as an indicator of gait abnormalities like limb dystonia or unsteady gait or tip-toe gait, all of them present in stroke patients [25, 29] and is consistent with a lesion affecting primary motor cortex [30]. However, we could not detect any differences in the other parameters studied. Previous studies have described differences in hind stride length and base width after transient MCAO in rats [27] but only at later times after ischemia (later than 14 days). Interestingly, we did not observe any differences using this test after ACA occlusion, as expected from lesions affecting structures such as frontal cortex (orbital and frontal association cortex), anterior cingulate cortex and supplementary motor cortex [21, 22].

Similar model-dependent results were obtained when anxiety-like behavior was analyzed with the elevated plus maze. As expected from previous studies [31], we observed a decrease in the time spent in open arms and in closed arm entries after left pMCAO at early times after ischemia, but not after left pACAO, suggestive of higher anxiety levels in the first one. Since several studies have described an association between anxiety symptoms and right hemispheric lesions in patients [23], we decided to performed cerebral ischemia in the opposite hemisphere in both models. Thus, we found anxious-like behavior after either right or left pMCAO but not after right or left pACAO. These results demonstrate the absence of a hemispheric effect on anxiety neurobehaviour after ischemia in CD1 mice. Although anxiety behaviour in mice after left or right MCAO has been reported [32], this is the first time that it is demonstrated at early times post-pMCAO. This is also the first report exploring ACAO in mice and demonstrating the lack of anxiety in this model.

Ischemic stroke and depression are frequently related. A high proportion of stroke patients develop mood disorders, being depression the most common neuropsychiatric condition (around 33% of post-stroke patients) [33]. It has been described that severity of depression depends on the hemisphere affected in humans although the studies are controversial [34, 35]. Regarding animal models, there is a lack of studies in the literature linking depression-like behavior with the hemisphere infarcted. Therefore, we explored depressive-like behavior after pMCAO or pACAO models in the two brain hemispheres of CD1 mice. Interestingly, our results show an increase in immobility time in both models when the occlusion was performed in left arteries but not when right hemisphere was affected. To our knowledge, this is the first evidence of hemisphere-dependent differences in depressant-like behaviour after experimental ischemia in mice. These data outline the importance of the experimental design when it comes to study functional deficits and provide an excellent tool to study stroke-induced depression at the preclinical level, addressing an unmet need in stroke studies.

In conclusion, we have demonstrated the applicability of several neurobehavioral tests, not commonly used in experimental stroke, which are able to detect neurological and psychological impairments early after ischemia in CD1 mice. The need to develop sensitive tests to neurological damage that will be able to characterize the effects of drugs at initial times after ischemia makes these results of great interest. We also highlight the need to take into account the territory affected by arterial occlusion as well as the hemisphere where brain damage occurs. All these findings will help scientific community to improve the experimental design in order to achieve better results in translational investigation of stroke.
